# Empty Follicle Syndrome: Current Therapeutic Approaches and the Role of Triggering Agents in Assisted Reproductive Technology

**DOI:** 10.3390/medsci14030369

**Published:** 2026-07-02

**Authors:** Sofoklis Stavros, Athanasios Zikopoulos, Stefanos Dafopoulos, Nektaria Zagorianakou, Efthalia Moustakli, Anastasios Potiris, Ismini Anagnostaki, Theodoros Karampitsakos, Konstantinos Dafopoulos, Peter Drakakis

**Affiliations:** 1 Third Department of Obstetrics and Gynecology, University General Hospital “ATTIKON”, Medical School, National and Kapodistrian University of Athens, 12462 Athens, Greece; apotiris@med.uoa.gr (A.P.); theokarampitsakos@hotmail.com (T.K.); pdrakakis@hotmail.com (P.D.); 2 Department of Reproductive Medicine and Surgery, University College London Hospitals NHS Foundation Trust, 235 Euston Road, London NW1 2BU, UK; thanzik92@gmail.com; 3 University General Hospital of Patras, 26504 Rio, Greece; stefanosntf2001@gmail.com; 4 Department of Nursing, School of Health Sciences, University of Ioannina, 4th Kilometer National Highway Str. Ioannina-Athens, 45500 Ioannina, Greece; zagorianakou@uoi.gr (N.Z.); ef.moustakli@uoi.gr (E.M.); 5 Medical School, National and Kapodistrian University of Athens, 11528 Athens, Greece; isanagnostaki3@gmail.com; 6 Department of Obstetrics and Gynaecology, Faculty of Medicine, School of Health Sciences, University of Thessaly, 41110 Larissa, Greece; kdafop@uth.gr

**Keywords:** final oocyte maturation, LHCGR, ovarian stimulation protocols, GnRH agonist, hCG pharmacokinetics, dual stimulation protocol, cumulus–oocyte complex, oocyte retrieval failure, periovulatory signaling cascade

## Abstract

The hallmark feature of empty follicle syndrome (EFS) is failure to retrieve oocytes from apparently mature follicles despite adequate ovarian stimulation and appropriate ovulation triggering. Although considered uncommon, with a reported prevalence ranging from 0.2% to 7%, EFS may have a profound clinical and psychological impact and can recur in assisted reproductive technology (ART) cycles. Modern classification systems divide EFS into genuine and false forms. Genuine EFS is potentially associated with intrinsic abnormalities involving luteinizing hormone/choriogonadotropin receptor (LHCGR) signaling, oocyte competence, and cumulus–oocyte interaction, whereas false EFS is primarily attributed to pharmacokinetic or pharmacodynamic factors resulting in inadequate trigger exposure. Borderline EFS represents a third phenotype characterized by incomplete or partial impairment of final oocyte maturation. This review examines the pharmacodynamics of ovulation-triggering agents, including human chorionic gonadotropin (hCG), gonadotropin-releasing hormone (GnRH) agonist protocols, and dual-trigger strategies, and their roles in regulating final oocyte maturation. The molecular aspects of periovulatory signal transduction and the mechanisms of LHCGR activation, epidermal growth factor (EGF)-like pathways, and meiotic resumption in relation to EFS etiopathogenesis will be described. The impact of patient-dependent conditions like obesity, poor ovarian reserve, polycystic ovary syndrome (PCOS), and pituitary response on trigger response will be assessed. New approaches like post-trigger monitoring of hormones and rescue treatment with gonadotropins represent a valuable method for avoiding cycle cancellation in patients at risk. Overall, EFS is increasingly regarded not as a single disorder but as a heterogeneous spectrum of periovulatory dysfunction arising from pharmacological, endocrine, and intrinsic ovarian factors that impair completion of final oocyte maturation.

## 1. Introduction

In the context of assisted reproductive technology (ART), the empty follicles syndrome (EFS) occupies a special place. While EFS is relatively rare, it represents a clinically significant complication that most reproductive specialists performing oocyte retrieval are likely to encounter, with implications extending beyond immediate cycle failure. An inability to retrieve oocytes from seemingly mature follicles following proper ovarian stimulation and follicular maturation is what defines EFS. Over 40 years of studying this condition, no consensus has been reached as to its underlying mechanisms and methods of prevention [1,2].

Reported prevalence varies widely across studies, ranging from approximately 0.2% in unselected in vitro fertilization (IVF) populations to nearly 7% in cohorts enriched for advanced maternal age and diminished ovarian reserve [3,4]. The heterogeneity in reported prevalence rates is likely attributable to both underlying biological variability and the absence of standardized diagnostic criteria across centers. Whereas some authors have considered oocyte retrieval to be abnormally low even when follicular development appears to be normal, others have defined EFS to include a complete absence of oocytes in all the retrieved follicles [5,6].

Contemporary classification recognizes three overlapping phenotypic forms of EFS, each characterized by distinct pathophysiological mechanisms and clinical consequences. False EFS is the most common phenotype and is typically characterized by inadequate pharmacological stimulation at the time of oocyte retrieval [7,8]. Reasons behind it include rapid clearance of the exogenous human chorionic gonadotropin (hCG), drug pharmacodynamics changes in people with high body mass index (BMI), medication that is either past its expiration date or stored improperly, and improper self-injection of hCG [9,10]. At the moment of oocyte retrieval, the level of luteinizing hormone (LH) or hCG is below the required limit. Nevertheless, genuine EFS does occur and appears to result from intrinsic failure of the final oocyte maturation process [7].

Dysregulation of oocyte–cumulus signaling and pathogenic variants affecting zona pellucida (*ZP*) genes or luteinizing hormone/choriogonadotropin receptor (LHCGR) have been proposed as contributory mechanisms; however, the molecular basis of these abnormalities remains incompletely characterized and markedly heterogeneous [11,12,13]. The term borderline EFS has been used to describe cycles with disproportionately low oocyte recovery relative to the number of mature follicles aspirated; however, standardized diagnostic criteria have yet to be established. This phenotype shares molecular similarities with genuine EFS and may respond to similar therapeutic approaches, although it does not represent classical EFS in the strict sense [14,15].

All of these differences have clinical implications as the treatment methods vary greatly from one phenotype to another. Optimization of drug administration, dosing adjustments in case of high risks, as well as hormonal surveillance after the trigger, can be employed in order to reduce EFS that occurs in such cases [16,17]. Conversely, genuine EFS often necessitates different triggering approaches, adjustments to treatment protocols in subsequent cycles, or, occasionally, consideration of oocyte donation [18].

Accordingly, individualized trigger selection has become increasingly important for optimizing ovarian stimulation outcomes in high-risk patients. hCG, which directly activates LHCGR to reproduce the physiological LH surge, remains the conventional reference trigger in both urinary and recombinant formulations [19,20]. Gonadotropin releasing hormone (GnRH) agonist triggers, which induce an endogenous gonadotropin surge through pituitary stimulation, have substantially reduced the risk of ovarian hyperstimulation syndrome (OHSS) in appropriately selected patients with preserved pituitary responsiveness [21,22,23]. Dual-trigger strategies combining a GnRH agonist with low-dose hCG are increasingly applied in patients at elevated risk for EFS or impaired oocyte maturation. In addition, newer agents acting further upstream within the HPG axis, such as kisspeptin, may provide a more physiological ovulatory response while potentially minimizing excessive ovarian stimulation, although clinical experience remains limited [24,25,26].

Trigger selection is further complicated by many patient-level characteristics. Obesity, particularly when BMI exceeds 30 kg/m^2^, alters the volume of distribution and apparent serum concentration of hCG, potentially affecting LHCGR activation thresholds at the granulosa cell level [10,27]. PCOS, diminished ovarian reserve, and advanced maternal age can all alter follicular response to a particular trigger dosage [28]. In addition to impairing pituitary responsiveness to GnRH agonist triggering, previous regimens using prolonged combination oral contraceptive priming or lengthy exposure to GnRH antagonists may potentially increase the risk of false EFS through mechanisms apart from obesity-related pharmacokinetic effects. As a result, the combination of patient phenotype, endocrine response, and previous cycle history is playing an increasingly important role in trigger selection [29,30,31,32].

This review investigates the idea that EFS represents uneven failure of the final oocyte maturation signal at the level of oocyte–cumulus communication, LHCGR-mediated granulosa cell signaling, or both. The idea that trigger selection should be customized based on patient phenotype and biological risk factors is supported by these processes. Therefore, biomarker-guided trigger techniques combined with post-trigger hormonal monitoring in certain individuals may help avoid and control EFS. The molecular physiology of final oocyte maturation, pharmacological triggering techniques, post-trigger monitoring, and new therapeutic methods for the treatment of EFS are covered in the sections that follow.

## 2. Literature Search Strategy

The PubMed/MEDLINE, Scopus, and Google Scholar databases were searched for relevant literature published between January 2008 and March 2026. Search terms included combinations of “empty follicle syndrome”, “EFS”, “oocyte maturation”, “final oocyte maturation”, “human chorionic gonadotropin”, “hCG”, “GnRH agonist trigger”, “dual trigger”, “double trigger”, “LHCGR”, “assisted reproductive technology”, and “ART”. Original clinical studies, systematic reviews, meta-analyses, case reports, and mechanistic studies relevant to EFS pathophysiology, trigger pharmacology, periovulatory signaling, and individualized IVF management were prioritized. Additional relevant publications were identified through manual screening of reference lists of selected articles. Due to the narrative nature of this review, formal systematic review methodology, risk-of-bias assessment, and quantitative synthesis were not performed.

## 3. Physiology of the Periovulatory Transition and the Rationale for Pharmacological Triggering

Comprehensive knowledge of final oocyte maturation physiology is fundamental to understanding the mechanisms of EFS and the basis of pharmacological triggering approaches in ART [19,33]. The endogenous LH surge initiates a coordinated series of molecular events that change a meiotically arrested oocyte and its surrounding follicular environment into a structure capable of ovulation and fertilization. Current triggering agents used in ART are designed to mimic or replace this physiological signal, although they differ substantially in pharmacodynamics and downstream biological effects [19,34,35].

### 3.1. Maintenance of Meiotic Arrest During Folliculogenesis

The oocyte is arrested at the diplotene phase of prophase I during most of folliculogenesis. High concentrations of cyclic adenosine monophosphate (cAMP) in the oocyte are responsible for arresting the oocyte. This arrest is supported by surrounding cumulus cells that provide cyclic guanosine monophosphate (cGMP) to the oocyte via gap junctions to inhibit phosphodiesterase 3A and prevent the breakdown of cAMP [36,37]. Therefore, the follicle acts as a well-coordinated unit, where the somatic compartment constantly maintains meiotic arrest. This communication network rapidly disassembles within hours after the LH surge, and the precision of this process is critical for successful retrieval of a fully mature oocyte [38,39].

### 3.2. LHCGR Expression and Initiation of the Periovulatory Cascade

The LHCGR, a family of receptors shared by LH and hCG, is a G protein-coupled receptor that is mostly expressed on theca cells. During the late follicular phase, follicle-stimulating hormone (FSH) activates this receptor on mural granulosa cells [34]. Follicular response to the periovulatory surge depends on the induction of LHCGR on granulosa cells; regardless of the severity of the surge, the dominant follicle cannot react effectively in the absence of sufficient receptor expression. Therefore, a phenotype that is clinically identical to insufficient triggering may result from defective LHCGR induction or reduced downstream receptor signaling, which has been linked to true types of EFS [40,41,42]. LHCGR activation primarily engages the Gαs/cAMP/protein kinase A (PKA) signaling pathway, although additional signaling through Gαq/phospholipase C and β-arrestin pathways also contributes. Increased granulosa-cell cAMP initiates a coordinated transcriptional program involving sequential downstream signaling events [43].

### 3.3. EGF-like Signaling and Cumulus Expansion

Rapid induction of the EGF-like peptides amphiregulin (AREG), epiregulin (EREG), and betacellulin (BTC) in mural granulosa cells is one of the initial reactions to LHCGR activation [44,45]. Because LHCGR expression in cumulus cells is restricted, these ligands operate in a paracrine manner on EGF receptors produced by cumulus cells and serve as crucial amplifiers of the LH signal. According to experimental data, oocyte maturation and cumulus growth are hampered when this EGF-like signaling network is disrupted [46,47].

Activation of the epidermal growth factor (EGF) receptor in cumulus cells induces phosphorylation of extracellular signal-regulated kinase 1/2 (ERK1/2), the principal downstream effectors of the MAPK3/1 signaling pathway, and upregulates hyaluronan synthase 2, prostaglandin-endoperoxide synthase 2 (PTGS2/COX2), and pentraxin 3, thereby promoting formation of the hyaluronan-rich extracellular matrix characteristic of cumulus expansion. The MAPK3/1 pathway is a central mediator of the periovulatory response, coordinating cumulus expansion, ovulatory gene expression, and the progression of oocyte maturation. Concurrently, ERK-mediated phosphorylation of connexin 43 reduces gap-junction communication between cumulus cells and the oocyte, which in turn reduces cGMP transport into the oocyte [47,48,49].

### 3.4. Meiotic Resumption and Oocyte Maturation

Reduction of intra-oocyte cGMP activates phosphodiesterase 3A (PDE3A), leading to degradation of cyclic adenosine monophosphate (cAMP). The resulting decline in cAMP relieves meiotic arrest and promotes activation of the cyclin B/CDK1 complex, also known as maturation-promoting factor (MPF). This initiates germinal vesicle breakdown, meiotic spindle formation, progression through metaphase I, and subsequent arrest at metaphase II, the stage associated with fertilization competence [50,51].

Granulosa cells, cumulus cells, and the oocyte itself must communicate in a highly synchronized manner for these processes to occur. Even if follicular development and triggering appear sufficient, disruption at any level of this communication network may hinder the maturation process and contribute to EFS abnormalities [52,53].

### 3.5. Ovulation, Luteinization, and Follicular Remodeling

The follicle is prepared for ovulation and luteinization during the last stage of the periovulatory cascade. Steroidogenic acute regulatory protein (StAR) and CYP11A1 are upregulated to boost progesterone production, while tissue plasminogen activator and matrix metalloproteinases aid in follicular wall remodeling. In parallel, MAPK-dependent periovulatory gene expression contributes to follicular rupture and luteinization. Following cumulus enlargement and the loss of integrin-mediated adhesion, the cumulus–oocyte complex separates from the mural granulosa layer [41,54,55].

Despite seemingly developed follicles, follicular aspirates may not contain a viable cumulus–oocyte complex if this last detachment fails. Thus, as Table 1 summarizes, each stage of this cascade indicates a possible mechanistic point of failure in EFS.

### 3.6. Physiological Basis of Pharmacological Triggering in ART

The aspects of the periovulatory cascade that have special relevance to pharmacological triggering in ART include precise timing of events and signaling by threshold levels of ligand binding [35]. The interval between the LH surge and ovulation is approximately 36–40 h; therefore, oocyte retrieval in stimulated cycles is carefully timed to occur close to ovulation. Improper trigger timing may lead either to retrieval before complete oocyte maturation or to premature ovulation [56,57,58]. Secondly, LHCGR stimulation is believed to require sustained receptor exposure to ligand concentrations above a critical threshold. Receptor engagement should be maintained continuously for several hours in order to facilitate full maturation [59,60].

The physiological factors described above account not only for the differences in the efficacy of triggering methods but also for the biological effects of the resulting gonadotropin peak [61]. The prolonged duration of action of urinary and recombinant hCG is attributed to their longer half-lives and higher affinity for the luteinizing hormone receptor [62]. In contrast, GnRH agonist triggering induces a transient surge of LH and FSH that closely resembles the physiological mid-cycle gonadotropin surge [22].

### 3.7. Mechanistic Implications for EFS

Furthermore, this physiological basis also helps in understanding why certain groups of patients might be prone to EFS. Even in cases where high levels of LH are considered sufficient, the natural gonadotropin flare generated due to the stimulation of a GnRH agonist is insufficient in sustaining sufficient activation of LHCGRs in severely suppressed pituitary systems [63,64,65]. Expanding the volume of distribution may lower serum hCG concentrations below the threshold needed for sustained receptor occupancy in patients with high BMI.

Lastly, even optimally timed and dosed pharmacological triggers may be insufficient to reproduce the physiological maturation cascade in individuals carrying LHCGR mutations or exhibiting inherent deficiencies in EGF-like signaling pathways [36,66]. These unique processes may require various therapy methods, as they most likely correspond to physiologically diverse subtypes of EFS.

## 4. Classification, Pathophysiology, and Risk Factors of EFS

The clinical heterogeneity of EFS has complicated both its diagnosis and management. EFS was typically defined as an uncommon and poorly understood failure of final oocyte maturation in earlier publications, which generally treated it as a separate entity. However, there are many phenotypes with different pathophysiological causes, prognostic consequences, and therapeutic considerations, according to available data [1,67].

### 4.1. False EFS

The most common and perhaps avoidable version of the condition is false EFS. Usually defined as blood hCG levels below 10 mIU/mL or an LH spike below 15 IU/L after GnRH agonist treatment, it is characterized by the failure of oocyte retrieval in the presence of insufficient circulating concentrations of the triggering agent at the moment of retrieval [18,68]. Therefore, rather than intrinsic follicular malfunction, the underlying mechanism reveals inadequate pharmacological signaling.

Potential causes include pharmaceutical factors such as expired medications, improper storage, incorrect formulation, or drug substitution. Administration errors, particularly during self-administration of hCG, persist despite improvements in injectable device technology [69].

In other instances, the levels of serum triggers are still inadequate despite proper administration of the drug. The most extensively studied scenario concerns altered pharmacokinetics in patients with elevated BMI, potentially attributable to reduced serum hCG concentrations and increased volume of distribution. Other possible reasons include changes in the glycosylation pattern of hCG and faster hCG metabolism [70,71,72].

False EFS is observed following GnRH agonist triggering. As this method uses stimulation of endogenous pituitary gonadotrophins, any profound suppression of the pituitary gland or deficiency in gonadotrophin reserves can affect the endogenous flare response. This phenomenon is observed in women suffering from hypothalamic amenorrhea, those with low initial LH levels, patients receiving long-term oral contraceptives, or those treated with GnRH antagonists [73,74]. The underlying defect appears to involve inadequate pituitary responsiveness rather than impaired drug administration.

### 4.2. Genuine EFS

The inability to retrieve oocytes even when there appears to be adequate administration of the trigger and high blood levels of hormones that exceed established thresholds defines EFS [75]. In this scenario, the patient may have had proper triggering and hormone treatment, but the follicle aspiration still fails to yield oocytes. Given that the cause may not be related to the drug signaling process but rather inherent to the process itself, the condition is rarer and harder to treat than pseudo EFS [18,31].

The molecular underpinnings of true EFS are still diverse and poorly understood. While functional receptor polymorphisms may contribute to interindividual heterogeneity in ovarian response, inactivating mutations affecting the LHCGR have been found in family instances of recurrent EFS [2,76]. Cumulus–oocyte complex integrity problems and recurring EFS symptoms have also been linked to variations in *ZP* genes, including *ZP1*, *ZP2*, and *ZP3* [77,78,79].

Altered downstream periovulatory signaling may also contribute to this condition. Cumulus expansion and oocyte release are hindered in experimental animals with compromised AREG, EREG, BTC, or EGFR-mediated signaling [49]. Rapid follicular atresia or premature luteinization may also play a role in some cases, producing follicles that appear morphologically mature even in the absence of a viable cumulus–oocyte complex during retrieval. Women with repeated low-yield or maturation-defective cycles have also been shown to have intrinsic oocyte abnormalities, such as mitochondrial dysfunction, meiotic spindle defects, and poor cytoplasmic maturation [33,80,81].

Repeating the same stimulation and triggering protocols may provide limited benefit, and genuine EFS remains difficult to manage. Modification of trigger selection, use of multiple or double trigger protocols, optimization of ovarian stimulation regimens, and consideration of oocyte donation in refractory patients are some of the suggested techniques [82,83]. Individuals with recurrent disease or familial history may benefit from targeted genetic testing and counseling.

### 4.3. Bordeline EFS

Although diagnostic criteria are still varied and the phenotype has not yet attained the same diagnostic stability as false or true EFS, borderline EFS has garnered growing clinical attention [2]. In general, it describes cycles in which oocyte recovery is abnormally low relative to the number of mature follicles aspirated [84]. Although threshold definitions vary, retrieval from fewer than approximately 50% of mature follicles is frequently used as a practical reference point, particularly when accompanied by low oocyte maturation rates [35,85].

Mechanistically, rather than total trigger failure, borderline EFS seems to represent partial disruption of the final oocyte maturation cascade. The endocrine signal induced by triggering may produce heterogeneous or subthreshold activation of periovulatory signaling pathways, thereby contributing to variable follicular responses within the same cycle [15,18,86]. Therefore, pharmacological insufficiency and intrinsic follicular dysfunction may overlap in borderline EFS, which shares characteristics with both false and true EFS.

Clinically, hCG dosage optimization, dual trigger protocols, post-trigger hormonal monitoring with rescue intervention when necessary, and other methods utilized in recurrent or high-risk cycles are frequently effective for individuals with questionable EFS [26,87].

### 4.4. Risk Factors

Although their proportional contribution changes depending on the underlying mechanism involved, risk factors for EFS significantly overlap across the various phenotypes. Table 2 summarizes the main risk variables identified in the literature.

Several factors may interact via multiple biochemical signaling pathways. For instance, obesity and PCOS often coexist and can contribute to heterogeneous follicular maturation linked to borderline EFS. Obesity may also influence trigger pharmacokinetics and serum hCG exposure, potentially affecting the efficacy of final oocyte maturation; however, a direct causal relationship between obesity and EFS has not been conclusively established [88,89]. Due to decreased reproductive potential and fewer opportunities for repeated treatment efforts, advanced maternal age and lower ovarian reserve frequently coexist and may exacerbate the clinical effects of unsuccessful oocyte retrieval [90,91].

Patients with several coexisting risk factors may benefit from individualized trigger selection in subsequent cycles and more stringent endocrine monitoring, given their potentially elevated risk of recurrent EFS [19,92].

Because treatment approaches vary based on the predominant underlying mechanism, this categorization paradigm also has significant therapeutic consequences. While true EFS may involve more extensive alteration of triggering techniques and evaluation of underlying biological dysfunction, false EFS simply demands improvement of pharmacological signaling. Individualized treatment including aspects of both techniques, is often necessary for borderline EFS [2,93].

**Table 2:** Principal risk factors associated with different phenotypes of EFS and their proposed mechanistic basis.

Risk Factor	Predominant EFS Phenotype	Proposed Mechanistic Basis
Advanced maternal age (>38 years) [81,90,91]	Genuine, borderline	Reduced oocyte competence, altered periovulatory signaling responsiveness, and accelerated follicular depletion.
Diminished ovarian reserve (low AMH, low AFC) [3,6,82]	Genuine, borderline	Reduced follicular cohort with diminished cumulative periovulatory signaling capacity
Obesity (BMI > 30 kg/m^2^) [10,27,72]	False	Altered hCG pharmacokinetics and reduced serum trigger concentrations
PCOS [73,88,89]	Variable	Heterogeneous follicular maturation and altered endocrine responsiveness influencing trigger efficacy
Prior EFS or previous low-maturation cycle [7,75,93]	All phenotypes	Increased recurrence risk in susceptible patients
Profound pituitary suppression (prolonged GnRH antagonist exposure or extended COC priming) [29,65,68]	False EFS with GnRH agonist trigger	Inadequate endogenous LH surge following GnRH agonist administration
*LHCGR* or *ZP* gene variants [3,6,53]	Genuine	Intrinsic disruption of periovulatory signaling or cumulus–oocyte complex integrity
Endometriosis with diminished ovarian reserve [94,95,96]	Genuine, borderline	Combined effects of impaired ovarian reserve and altered follicular microenvironment

## 5. Triggering Agents: Mechanisms, Pharmacology, and Pharmacokinetics

To prevent EFS, pharmacological stimulation of ultimate oocyte maturation is a crucial stage in IVF therapy. Oocyte maturation and retrieval results may be greatly impacted by the wide variations in endocrine dynamics, pharmacokinetic characteristics, and dependency on endogenous pituitary function among available triggering agents [33,35,86].

### 5.1. Urinary hCG

The first extensively utilized pharmacological trigger for ultimate oocyte maturation was urinary hCG (u-hCG), which is still often used in many IVF protocols. The biological activity of hCG is standardized in international units (IU), most commonly within the range of 5000–10,000 IU [14]. The prolonged elimination half-life of u-hCG (approximately 24–36 h) results in sustained LHCGR activation following direct receptor binding [97,98].

In addition to consistently promoting the completion of the periovulatory maturation cascade, the long-term luteotropic impact of u-hCG increases the risk of OHSS, especially in high responders. Advantages include extensive clinical experience and flexibility in dose escalation, particularly in patients with elevated BMI. Limitations include extended corpus luteum stimulation and increased batch-to-batch variability relative to recombinant formulations [24,99].

### 5.2. Recombinant hCG

Recombinant hCG (r-hCG) was developed to reduce the batch-to-batch variability and potential contamination concerns associated with urinary-derived formulations. The standard dose of 250 μg is considered approximately equivalent to 6500 IU of urinary hCG (u-hCG). Unlike urinary-derived preparations, r-hCG is a structurally well-defined molecule with consistent glycosylation characteristics, and its biological activity is standardized by mass rather than expressed in international units [97,100].

Both r-hCG and u-hCG exhibit similar receptor-binding properties and, owing to their comparably prolonged elimination half-lives, promote sustained LHCGR activation. The general pharmacodynamic profile is essentially the same, although some studies suggest a somewhat quicker clearance with the recombinant formulation [97,101,102].

From a clinical perspective, r-hCG is associated with superior batch consistency, lower rates of injection-site reactions, and removal of impurities associated with urinary-derived preparations [103]. In standard IVF practice, r-hCG and u-hCG yield broadly comparable oocyte maturation and pregnancy outcomes in unselected populations; therefore, selection is frequently determined by cost and local availability rather than significant differences in effectiveness [97,104].

The distinction between these agents may become more important in specific patient populations, especially in women with high BMI. The fixed-dose administration of r-hCG may produce suboptimal serum concentrations in individuals with obesity, potentially compromising adequate pharmacological signaling at triggering [10,70]. On the contrary, in individuals who are thought to be more susceptible to false or borderline EFS, u-hCG allows for more flexibility in dosage escalation [15,105].

### 5.3. GnRH Agonist Trigger

A radically different pharmacological approach to ultimate oocyte maturation is represented by the GnRH agonist trigger. This approach stimulates pituitary GnRH receptors to induce an endogenous gonadotropin surge rather than directly administering an exogenous LHCGR ligand [22,106]. A single bolus dose of a GnRH agonist, usually triptorelin 0.2 mg, leuprolide 1–2 mg, or buserelin 0.5 mg, is administered in GnRH antagonist cycles to displace the antagonist from pituitary receptors and cause the abrupt release of LH and FSH from preformed pituitary storage. Serum gonadotropin concentrations typically reach their peak in about 4 h and then start to decrease over the next 24 to 36 h [64,107,108].

The endocrine profile produced by GnRH agonist treatment is more similar to the physiological mid-cycle spike than hCG-based triggering [22]. Crucially, both LH and FSH components are present in the endogenous flare, which may aid in cumulus enlargement and the completion of periovulatory signaling pathways [109]. GnRH agonist triggering significantly lowers the risk of severe OHSS because the luteotropic stimulation provided to the corpus luteum is far shorter than that associated with hCG [22]. Nevertheless, the shortened luteal phase support associated with this approach commonly leads to luteal insufficiency, thereby requiring intensive luteal supplementation or adoption of a freeze-all strategy [110].

The dependency of GnRH agonist triggering on sufficient pituitary response is its main drawback. Despite receiving the right trigger, patients with severe pituitary suppression may not produce a significant endogenous gonadotropin surge [111,112]. Approximately 12 h after triggering, serum LH ≥ 15 IU/L and progesterone > 3 ng/mL are commonly used indicators of an adequate response. Suboptimal endocrine response despite appropriate drug administration may impair activation of the maturation cascade, thereby contributing to false EFS through endocrinological rather than pharmacokinetic mechanisms [68,112].

Individuals with hypothalamic amenorrhea, prolonged pretreatment with combination oral contraceptives, prolonged exposure to GnRH antagonists, and low baseline LH concentrations at the beginning of ovarian stimulation are at higher risk [113,114,115].

### 5.4. Recombinant LH and Kisspeptin

An alternate direct LHCGR agonist that may more nearly mimic the normal periovulatory surge than hCG is recombinant LH (r-LH). r-LH produces a more physiological exposure profile and may potentially lower the risk of OHSS due to its shorter elimination half-life, which is around 10–12 h [116]. However, its routine clinical use has been limited by the high doses required to achieve adequate final oocyte maturation, typically ranging from 15,000 to 30,000 IU. Currently, r-LH has not supplanted hCG- or GnRH agonist-based triggering techniques in conventional IVF therapy, and it is still mostly limited to certain clinical circumstances and research contexts [117].

Kisspeptin is another upstream triggering method, which has a completely different mechanism of action. Through activation of KISS1R receptors on hypothalamic GnRH neurons, kisspeptin induces endogenous GnRH release followed by pituitary secretion of LH and FSH [118]. Consequently, the endocrine milieu retains the natural hierarchical order of the hypothalamus-pituitary-gonadal axis and appears more physiological than with hCG or GnRH agonist triggering [61,119].

Clinical trials, particularly those conducted in the United Kingdom, have demonstrated low OHSS incidence together with favorable oocyte maturation and retrieval outcomes in both oocyte donors and patients with PCOS. Although kisspeptin is still being studied, the biological rationale for its use is strong, and clinical utilization is likely to be common in the near future [119,120,121].

### 5.5. Pharmacokinetic Considerations in Obesity

The relationship between adiposity and trigger-drug pharmacokinetics deserves special consideration, as it represents a potentially modifiable factor that may influence trigger effectiveness in IVF treatment. Obesity has been associated with alterations in hCG pharmacokinetics and lower serum hCG concentrations following standard trigger [122,123].

Increased body mass may expand the apparent volume of distribution of hCG, potentially resulting in lower circulating hormone concentrations after administration. Consequently, a fixed dose of hCG may generate lower peak serum concentrations (Cmax) and altered systemic exposure in women with elevated BMI [10,124,125]. Pharmacokinetic studies have reported lower serum hCG levels following standard trigger administration in obese women compared with normal-weight women receiving the same dose. Although these findings suggest a potential mechanism for suboptimal trigger response in some patients, current evidence does not establish obesity as a direct cause of EFS [10].

Other metabolic processes could potentially be involved. Granulosa-cell steroidogenesis and LHCGR expression have been demonstrated to be influenced by insulin resistance and prolonged hyperinsulinemia in experimental settings. Consequently, obesity-related metabolic alterations may influence follicular responsiveness to gonadotropin signaling, although the therapeutic relevance of these changes beyond pharmacokinetic dilution has not yet been fully established [126,127,128].

Dual trigger procedures, hCG dosage escalation, and regular post-trigger hormonal monitoring about 12 h after injection are possible management techniques. Although alternative dosing strategies based on weight or body surface area have been proposed, most current regimens still use BMI-based dose adjustment instead of individualized pharmacokinetic models [83,129].

## 6. Dual Trigger, Double Trigger, and Post Trigger Monitoring with Rescue Protocols

Tο customize therapy and lower the risk of EFS, combination triggering techniques and post-trigger hormonal monitoring have become more common. This is because no single triggering strategy is ideal for all patient types. The main modern methods for optimizing final oocyte maturation in high-risk patients include rescue interventions, dual trigger protocols, and double trigger tactics [19,86,130].

### 6.1. Rationale for Combined Triggering

The dual trigger produces both an endogenous gonadotropin flare and persistent LHCGR activation by concurrently administering a GnRH agonist and a lower dose of hCG. This method minimizes some of the drawbacks of each technique alone while attempting to replicate important physiological aspects of the normal periovulatory surge [87,131,132]. While the hCG component offers longer receptor stimulation and makes up for the potential for an insufficient pituitary response, the endogenous LH and FSH surge brought on by the GnRH agonist may assist cumulus enlargement and the completion of periovulatory signaling [131].

Relative to conventional hCG triggering, dual-trigger strategies using reduced hCG doses (approximately 1000–1500 IU urinary hCG or equivalent recombinant dosing) appear to preserve luteal support while minimizing OHSS risk [117,133].

There have been numerous reports over the last decade that confirm the superiority of dual triggering in the clinic. The randomized trials and observational data from normal responders show that there is no difference between hCG alone and dual triggering in terms of oocyte maturation rates, and the risk of OHSS is lower with the latter modality [134,135,136,137]. The benefits of dual triggering appear to be more pronounced in specific high-risk groups, including poor responders, patients with previous low oocyte maturation rates, and individuals with prior episodes of EFS. Evidence suggests that dual triggering enhances oocyte retrieval and metaphase II oocyte yield relative to hCG-only protocols in selected patients; however, inter-study heterogeneity persists, and the optimal hCG dosing strategy has yet to be determined [19,83,138].

### 6.2. The Double Trigger

Although commonly conflated in the literature, double-trigger and dual-trigger protocols constitute distinct therapeutic strategies and should be clearly differentiated. Double triggering is a procedure where the triggering drugs are administered successively over a specific period of time, usually starting with a GnRH agonist and then followed by hCG after about 40 h, while the oocyte retrieval is conducted after some time delay. On the other hand, dual triggering entails administering both the GnRH agonist and hCG at the same time [35,83].

In patients who repeatedly show poor oocyte maturation despite apparently adequate trigger exposure, the underlying mechanism may reflect delayed completion of final oocyte maturation rather than failure of maturation initiation. Thus, prolonging the interval between trigger administration and oocyte retrieval while maintaining sustained periovulatory stimulation may permit completion of nuclear and cytoplasmic maturation [35,57,58].

Although fewer studies have been conducted on double triggers than dual triggering, double trigger protocols have been found to result in higher MII oocyte and fertilization rates in some patients [15]. Since the patient population already has optimized conventional or dual trigger treatments, current use is confined to patients with a history of low maturation rates or poor-quality oocytes. As such, double triggering is considered a secondary strategy instead of a primary one in IVF [83,117].

### 6.3. Selection of Triggering Strategies

Selection of the optimal triggering strategy should consider factors such as OHSS risk, predisposition to EFS, ovarian reserve, prior cycle response, BMI, and pituitary responsiveness [19,35,118].

In normal responding patients with no major risk of OHSS or history of issues in relation to triggering and insufficient oocyte maturation, conventional hCG triggering using either urinary or recombinant formulations remains a reasonable option. The proven effectiveness, simplicity, and years of clinical practice make it the recommended method in such cases [98,103,118].

The use of GnRH agonists as triggers is generally preferred in cases of high responders, particularly PCOS patients or women with a large number of antral follicles, where prevention of OHSS is the priority, and the decision to proceed to the “freeze-all” procedure is common practice [23,30,86]. Pituitary sensitivity is crucial for GnRH triggering and can be assessed based on basal LH levels before ovarian stimulation [68,114,116].

Patients who are thought to be at higher risk for poor maturation or false EFS—such as women who are obese, have reduced ovarian reserve, are older mothers, have had low-maturation cycles in the past, or have had EFS—are increasingly using dual triggering [26,83,94]. This approach is frequently used in patients with intermediate OHSS risk to optimize oocyte maturation while limiting excessive luteal stimulation. Dual trigger protocols are being used more often in many centers as customized approaches for patients who do not clearly fall into the traditional hCG- or GnRH agonist-only categories [24,83,117].

Despite previous optimization with conventional or dual trigger methods, the double trigger strategy is often reserved for certain individuals with recurrent poor oocyte maturation. In these situations, the goal of extended periovulatory stimulation and delayed retrieval time is to facilitate the completion of nuclear and cytoplasmic maturation [15,57,58].

Recombinant LH and kisspeptin have not yet been incorporated into standard clinical triggering algorithms and remain largely restricted to specialized tertiary-care or research settings.

### 6.4. Post-Trigger Hormonal Monitoring

While customized triggering methods have decreased the probability of insufficient final oocyte maturation, they cannot entirely rule out the possibility of either failure or inadequate response to the endocrine system [139]. The use of post-trigger hormone monitoring has thus become a crucial preventative measure in women who are at higher risk of EFS by enabling the detection of insufficient triggering prior to oocyte retrieval [7,105].

Hormonal assessment is typically performed approximately 12 h after triggering, when the physiological response is expected to have reached a relatively stable phase. The analytes that will be determined will depend on the mode of triggering [29]. In hCG-triggering, the serum concentration of hCG will be determined. The threshold concentration for hCG that has been commonly employed usually exceeds 100 mIU/mL. With the use of GnRH agonist triggering, the serum levels of LH and progesterone become more relevant, with threshold concentrations being >15 IU/L and >3 ng/mL, respectively [140,141].

In dual trigger protocols, both hCG- and pituitary-derived responses may be evaluated, although LH and progesterone measurements are often considered most clinically useful because they assess the adequacy of the endogenous gonadotropin flare [19].

Importantly, the hormonal thresholds currently used in clinical practice are based primarily on observational correlations with oocyte retrieval outcomes rather than rigorously validated biological cutoffs [142]. Accordingly, these values should be interpreted as practical clinical guides rather than absolute determinants of success or failure. Thresholds also vary among centres, and borderline results are best interpreted in the context of the individual patient’s overall clinical risk profile rather than in isolation [143]. The principal hormonal thresholds and their clinical interpretation are summarized in Table 3.

### 6.5. Rescue Protocols

Rescue intervention may stop cycle cancellation or the development of false EFS when post-trigger hormonal monitoring reveals an insufficient endocrine response [1,7,105]. The most popular rescue approach is to provide 10,000 IU of urinary hCG or its recombinant counterpart as soon as the insufficient response is identified. Instead of after the first trigger injection, oocyte retrieval is then delayed to around 36 h following the rescue dosage [65,144]. This strategy aims to synchronise retrieval timing with optimal cumulus–oocyte complex separation while ensuring sufficiently prolonged LHCGR activation to complete the periovulatory maturation cascade [35,56,58].

Rescue strategy implementation necessitates timely clinical re-evaluation, as well as adjustments to luteal support and retrieval timing. Administration of an extra hCG dosage, while typically successful, may slightly raise the risk of OHSS, especially in high responders who were previously treated with GnRH agonist triggering expressly to reduce this complication [23,25,86]. Alternative rescue strategies utilising an extra dosage of GnRH agonist have been suggested for these individuals, although there is currently little clinical data to support them [111,139].

**Figure 1:**
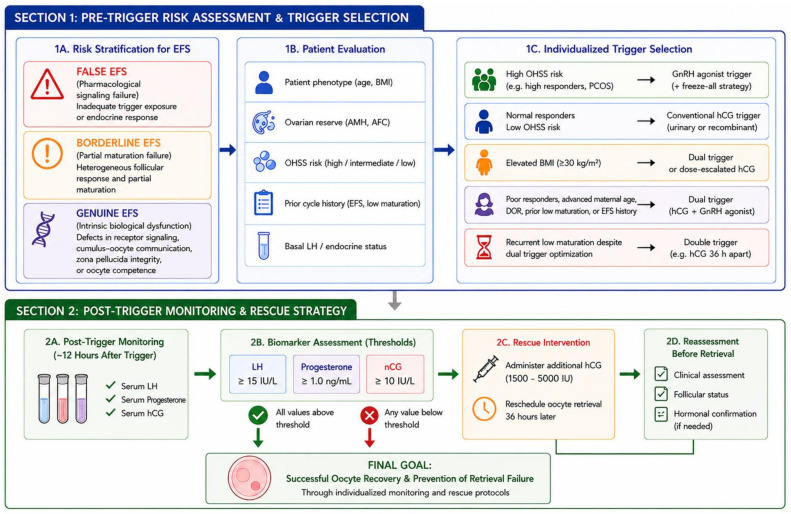
Individualized clinical algorithm for prevention and management of EFS during ART. Schematic overview of risk stratification, trigger selection, post-trigger hormonal monitoring, and rescue interventions used to reduce the risk of failed oocyte retrieval and optimize final oocyte maturation in patients undergoing IVF treatment.

Rescue interventions have demonstrated high success rates when implemented promptly in carefully selected patients. Observational studies report salvage rates of approximately 80–90%, with outcomes often comparable to those of initially successful triggering cycles [2,11,12,78,93]. The overall algorithm for pre-trigger risk stratification, individualized trigger selection, post-trigger hormonal monitoring, and rescue intervention is summarized in Figure 1.

## 7. Comparative Analysis of Triggering Agents

The pharmacological characteristics and clinical applications of the triggering strategies discussed above are comparatively summarized in Table 4A,B. The table incorporates each approach’s mode of action, endocrine profile, OHSS risk, implications for preventing EFS, main clinical indications, and significant drawbacks. This framework is designed to guide personalized therapeutic decision-making according to patient phenotype, ovarian response, previous cycle performance, and reproductive risk profile, rather than functioning as a fixed treatment algorithm [19,35,117].

This comparison yields some useful insights. First, since dual trigger methods have been more widely accepted, especially in patients with intermediate OHSS risk or prior triggering-related problems, the traditional binary distinction between hCG and GnRH agonist triggering has become increasingly supplemented by individualized and dual-trigger approaches. Dual triggering is increasingly being adopted as a practical intermediate strategy aimed at balancing adequate periovulatory signaling with reduced luteal overstimulation [83,87,138].

Second, depending on the underlying EFS phenotype, there are different relationships between the triggering approach and EFS risk. Pharmacological and endocrine variables play a major role in false EFS, which may be mitigated through individualized trigger selection, dosage optimization, dual triggering, and post-trigger endocrine monitoring. On the other hand, because genuine EFS reflects inherent problems within the oocyte maturation cascade rather than insufficient endocrine stimulation, it seems to be far less dependent on the triggering agent itself [7,33,93].

There are significant ramifications for patient counseling from this difference. Although modifying the triggering strategy may not prevent recurrent genuine EFS associated with underlying biological dysfunction, it may substantially reduce the risk of avoidable false EFS. Therefore, when recurrent EFS occurs despite apparently appropriate pharmacological therapy, broader etiological factors such as intrinsic follicular abnormalities or genetic predisposition should be considered [11,60,93].

There is significant variation in the quality of the data that supports the various triggering strategies. A comparatively large body of clinical research, including randomized and comparative trials, supports dual trigger techniques, GnRH agonist protocols, and conventional hCG triggering. In contrast, recombinant LH and kisspeptin are currently supported mainly by smaller clinical series and mechanistic studies, and their use is best considered within specialized or research-oriented settings [83,86,120].

## 8. Integrative Perspectives and Future Directions

### 8.1. EFS as a Heterogeneous Disorder of Final Oocyte Maturation

The idea that EFS is not a unique disease entity but rather a diverse clinical phenomenon is supported by current evidence [7,33,93]. The diverse manifestations of impaired final oocyte maturation occurring at different stages of the periovulatory signaling cascade may be reflected by false, genuine, and borderline EFS phenotype. Despite the adequate development of follicles, these abnormalities ultimately converge clinically through failure to retrieve mature oocytes, irrespective of their underlying mechanism [2,33,93]. Pharmacological, hormonal, and cellular dysfunction involving LHCGR signaling may represent one of the principal unifying mechanisms underlying this model [19,33,60].

Available evidence suggests that genuine EFS is predominantly linked to intrinsic defects involving receptor signaling, cumulus–oocyte interactions, ZP integrity, and oocyte competence, while false EFS is mainly associated with insufficient pharmacological exposure or suboptimal hormonal response [11,12,78,93]. Borderline EFS has been proposed as an intermediate phenotype characterized by heterogeneous follicular responsiveness and incomplete completion of final oocyte maturation within a given cycle [2,14,15].

This broader conceptual framework may help explain the marked variability in treatment response and clarify why EFS can occur despite apparently appropriate ovarian stimulation. Collectively, these observations support the view that EFS may be better interpreted as a spectrum of periovulatory dysfunction rather than a single disease entity [7,33,93].

### 8.2. Clinical Implications for Individualized Trigger Selection

Clinical treatment is significantly impacted by our growing understanding of the pathophysiology of EFS. Individual differences in the processes leading to EFS make a universal triggering method unlikely to be optimum across all patient groups. While patients with severe pituitary suppression may be more susceptible to insufficient endogenous gonadotropin release after GnRH agonist triggering, patients with obesity may predominantly have altered trigger pharmacokinetics and decreased serum hCG exposure. Recurrent EFS despite adequate hormonal responses may indicate intrinsic defects in the maturation cascade [10,68,93].

These findings provide credence to an increasingly customized method of trigger selection. In normal responders with low OHSS risk and no history of triggering-related problems, conventional hCG triggering is still quite successful. Provided that adequate pituitary responsiveness is preserved, GnRH agonist triggering is particularly beneficial in high responders and patients at increased risk of OHSS. Because dual trigger techniques combine endogenous gonadotropin release with continuous LHCGR activation, they are particularly helpful in individuals with intermediate-risk profiles, obesity, past poor maturation rates, borderline EFS, or prior unsuccessful retrieval efforts. In certain individuals with recurrent poor oocyte maturation, when delayed nuclear or cytoplasmic maturation is complete, double trigger techniques may offer extra benefit [23,83,117].

The growing emphasis on individualized trigger selection reflects the increasing clinical importance of patient phenotype, OHSS risk, ovarian reserve, BMI, pituitary function, and prior cycle response. In the absence of a universally accepted triggering algorithm, individualized trigger selection may be considered on the basis of patient characteristics, ovarian response, and previous treatment outcomes. However, many proposed approaches remain supported primarily by observational evidence and require further prospective validation. Table 5 summarizes potential trigger-selection approaches reported in the literature and should be interpreted as a conceptual framework rather than a validated clinical guideline [19,83,117].

Additionally, current evidence indicates that adequate pharmacological exposure alone does not always ensure successful oocyte retrieval. The occurrence of EFS in patients with documented serum hormone concentrations above accepted trigger-response thresholds supports the concept that downstream defects within the maturation cascade may contribute despite apparently sufficient triggering. Distinguishing these clinical scenarios is particularly important for patient counseling because optimization of the triggering strategy may reduce the likelihood of false EFS. Conversely, recurrent EFS is generally less responsive to pharmacological intervention and should prompt evaluation for intrinsic biological abnormalities [2,11,93].

### 8.3. Individualized Triggering and Post-Trigger Endocrine Monitoring

Moreover, the requirement for personalized treatments is highlighted by the rescue process and hormonal evaluation after trigger administration. With systematic monitoring approaches being used in fertility centers, such an approach may reduce avoidable false EFS cases through early detection of inadequate endocrine response before oocyte retrieval [7,105,139].

The possibility of detecting inadequate pharmacological exposure and/or insufficient pituitary response can be evaluated through serum hCG, LH, and progesterone measurements following trigger administration. Rescue hCG administration followed by delayed oocyte retrieval has demonstrated favorable cycle salvage rates in selected patients and may reduce the likelihood of cycle cancellation [65,68,129,144].

However, current hormonal cut-offs remain largely pragmatic rather than biologically validated and are primarily derived from observed clinical associations. Variability in assay methodology and institutional protocols further limits uniform interpretation of available evidence [61,117,142].

### 8.4. Current Limitations and Future Research Directions

Several important aspects of EFS prevention and management remain insufficiently understood. Considerable variability remains in the scientific literature regarding the optimal hCG dose for dual-trigger strategies. Similarly, further research needs to be conducted regarding the use of hormone cutoff levels following triggering [83,117,139].

Furthermore, the genetic mechanisms underlying recurrent genuine EFS remain incompletely defined. Current evidence suggests that only a minority of EFS cases can presently be attributed to identifiable genetic abnormalities, although pathogenic variants in *LHCGR* and *ZP* genes have been reported in selected familial cases. Available evidence supports the existence of additional molecular pathways influencing granulosa-cell function, oocyte competence, mitochondrial regulation, and periovulatory signaling [11,12,60,78]. Future trigger customization may further benefit from emerging approaches such as kisspeptin triggering in patients at high risk of OHSS. In addition, the effects of metabolic, inflammatory, and neuroendocrine processes on periovulatory signaling remain incompletely understood and represent an important area for future investigation [118,120,121].

Advances in the field increasingly support the replacement of fixed ovulation-triggering protocols with personalized approaches based on patient phenotype and clinical characteristics [19,93,117].

## 9. Conclusions

EFS continues to be a major challenge in assisted reproduction due to its multifactorial nature. The available literature supports the concept that false, genuine, and borderline EFS are distinct yet overlapping pathophysiological phenotypes characterized by impaired final oocyte maturation involving biochemical, endocrine, and intrinsic follicular dysfunction.

Several advances in trigger pharmacology, including GnRH agonist protocols, dual triggering, double-trigger approaches, and post-trigger hormonal monitoring, have proven effective in preventing false EFS and reducing failed oocyte retrieval due to avoidable causes. The importance of intrinsic defects in the periovulatory maturation sequence and the limitations of drug therapy can be seen biologically through repeated EFS occurrences.

Modern management of EFS increasingly relies on personalized trigger selection guided by phenotypic characteristics, previous cycle performance, endocrine responsiveness, and OHSS risk. Future research should extend beyond the classical hypothalamic–pituitary–ovarian axis and further investigate the role of metabolic, inflammatory, and endocrine modulators of final oocyte maturation. Particular attention should be given to insulin resistance, adipokine signaling, ovarian mitochondrial function, OS pathways, local cytokine networks, and steroidogenic regulation within the follicular microenvironment. The identification of endocrine and molecular biomarkers capable of predicting trigger responsiveness and EFS risk may facilitate the development of precision-medicine approaches and more individualized triggering strategies in ART.

## Figures and Tables

**Table 1:** Major molecular stages of the periovulatory maturation cascade and their potential relationship to EFS.

Stage of Maturation	Key Molecular Event	Potential Consequence if Disrupted
LHCGR induction on granulosa cells [34,42,43]	FSH-mediated receptor expression during the late follicular phase	Periovulatory surge cannot be adequately transduced; phenotype may resemble genuine EFS
LH/hCG binding and Gαs/cAMP/PKA activation [34,35,55]	Initiation of intracellular signaling within mural granulosa cells	Inadequate downstream signaling despite apparently adequate serum hormone concentrations
Induction of AREG, EREG, and BTC [44,45,47]	Paracrine propagation of the periovulatory signal to cumulus cells	Impaired cumulus expansion and persistence of meiotic arrest
Cumulus expansion and connexin 43 phosphorylation [38,49,52]	Disruption of cumulus–oocyte gap-junction communication	Persistent cGMP transfer prevents meiotic resumption
PDE3A activation and intra-oocyte cAMP hydrolysis [36,37,39]	Activation of maturation-promoting factor (MPF)	Germinal vesicle breakdown and meiotic progression fail to occur
StAR and CYP11A1 upregulation with extracellular matrix (ECM) remodeling [34,40,41]	Luteinization and follicular wall remodeling	Failure of cumulus–oocyte complex detachment resulting in empty follicular aspirates

**Table 3:** Commonly used post-trigger hormonal thresholds and their clinical interpretation in IVF cycles at increased risk for EFS.

Trigger Type	Measured Analyte	Typical Threshold at ~12 h Post-Trigger	Clinical Interpretation If Below Threshold
u-hCG or r-hCG [10,129,141]	Serum hCG	≥100 mIU/mL	Suggests inadequate pharmacological exposure; rescue hCG administration may be considered
GnRH agonist trigger [68,139,144]	Serum LH	≥15 IU/L	Suggests insufficient endogenous pituitary LH surge; rescue hCG administration may be required
GnRH agonist trigger [35,61,117]	Serum progesterone	>3 ng/mL	Low levels may indicate inadequate initiation of luteinization
Dual trigger [24,83,116]	Serum hCG	As above	Assesses adequacy of the endogenous agonist-induced gonadotropin response

**Table 4:** (**A**) Pharmacological and Endocrine Characteristics of Available Triggering Strategies. (**B**) EFS-Related Considerations and Limitations of Available Triggering Strategies.

(**A**)			
**Triggering Strategy**	**Primary Molecular Target**	**Endocrine/Surge Profile**	**OHSS Risk**
u-hCG [9,62,97]	Direct LHCGR agonist	Prolonged receptor stimulation; elimination half-life ~24–36 h	Moderate to high in high responders
R-hCG [97,102,103]	Direct LHCGR agonist	Prolonged receptor stimulation; elimination half-life ~24–36 h	Moderate to high in high responders
GnRH agonist trigger [23,30,35]	Pituitary GnRH receptor	Short endogenous LH/FSH surge approximating physiological profile	Very low
Dual trigger (GnRH agonist + low-dose hCG) [83,87,116]	Combined pituitary flare and direct LHCGR stimulation	Combined endogenous flare with sustained luteotropic support	Low to moderate
Double trigger [15,57,58]	Sequential pituitary flare followed by prolonged LHCGR activation	Extended periovulatory stimulation with delayed retrieval	Moderate
Recombinant LH [63,109,117]	Direct LHCGR agonist	Shorter and more physiological exposure profile; half-life ~10–12 h	Low
Kisspeptin [118,120,121]	Hypothalamic KISS1R receptor	Physiological endogenous GnRH-mediated surge profile	Low
(**B**)			
**Triggering Strategy**	**Implications for EFS**		**Principal Limitations**
u-hCG [10,19,101]	Effective for inducing final oocyte maturation; inadequate exposure may contribute to false EFS in selected patients.		Higher OHSS risk in high responders; variable absorption and exposure.
R-hCG [10,97,102]	Provides standardized LHCGR stimulation; lower serum exposure may occur in women with elevated BMI receiving fixed doses.		Higher OHSS risk in susceptible patients; limited dose flexibility.
GnRH agonist trigger [23,68,139]	May reduce trigger-related complications but can be associated with inadequate endogenous gonadotropin release in profoundly suppressed patients, potentially increasing false EFS risk.		Ineffective in severe pituitary suppression or very low baseline LH states.
Dual trigger (GnRH agonist + low-dose hCG) [83,87,132]	May enhance final oocyte maturation by combining endogenous gonadotropin release with sustained LHCGR stimulation; frequently considered in patients with previous low maturation or suspected trigger-related EFS.		Residual OHSS risk compared with GnRH agonist-only protocols.
Double trigger [15,57,82]	May improve maturation-defective phenotypes through prolonged periovulatory stimulation and extended oocyte maturation time.		Limited evidence base; increased protocol complexity.
Recombinant LH [63,109,117]	Potential alternative strategy when conventional triggering is unsuccessful, although supporting evidence remains limited.		High cost, limited availability, and insufficient clinical validation.
Kisspeptin [118,120,121]	Promising physiological trigger approach, but its role in EFS prevention or management remains unclear.		Investigational status and limited long-term clinical data.

**Table 5:** Proposed individualized trigger selection according to patient profile and clinical characteristics.

Clinical Scenario/Patient Profile	Potential Triggering Strategy	Rationale
Normal responder with low OHSS risk [97,102,117]	Conventional urinary or recombinant hCG	Reliable oocyte maturation with extensive clinical validation
High responder or PCOS with elevated OHSS risk [23,30,86]	GnRH agonist trigger ± freeze-all strategy	Markedly reduces risk of severe OHSS while maintaining adequate maturation
Obesity (BMI > 30 kg/m^2^) [10,125,129]	Dual trigger or modified hCG dosing may be considered	Compensates for altered hCG pharmacokinetics and reduced serum exposure
Advanced maternal age or diminished ovarian reserve [132,134,138]	Double trigger may be considered	May improve oocyte maturation and support heterogeneous follicular responsiveness
Previous low oocyte maturation or borderline EFS [83,87,131]	Double trigger may be considered	Combines endogenous gonadotropin flare with sustained LHCGR activation
Recurrent low maturation despite dual trigger [15,57,58]	Double trigger may be considered	Shorter and more physiological exposure profile; half-life ~10–12 h
Profound pituitary suppression or low baseline LH [68,139,144]	Conventional hCG trigger may be preferred over GnRH agonist alone	Reduces risk of inadequate endogenous gonadotropin surge
Previous false EFS [7,105,139]	Dual trigger with post-trigger hormonal monitoring	Minimizes risk of inadequate endocrine exposure and permits rescue intervention
Recurrent genuine EFS despite adequate hormonal response [11,12,93]	Individualized protocols; consider genetic evaluation	Pharmacological optimization alone may not overcome intrinsic biological dysfunction
Intermediate OHSS risk requiring balanced approach [83,87,116]	Dual trigger may be considered	Balances adequate maturation with reduced luteal overstimulation

## Data Availability

No new data were created or analyzed in this study.
